# The somatic care situation of people with mental illness

**DOI:** 10.1002/hsr2.226

**Published:** 2020-12-21

**Authors:** Ida Haussleiter, Barbara Emons, Knut Hoffmann, Georg Juckel

**Affiliations:** ^1^ Department of Psychiatry, LWL‐Institute of Mental Health Ruhr University Bochum Bochum Germany; ^2^ Department of Psychiatry, LWL University Hospital Bochum Ruhr University Bochum Bochum Germany

**Keywords:** cardiovascular risk factors, mental illness, physical health, somatic care, treatment motivation

## Abstract

**Background and Aims:**

People with mental illness have worse physical health and reduced life expectancy compared to the general population. Nevertheless, their medical care is often insufficient. The present study aimed to investigate the somatic status of people with mental illness with a focus on somatic diagnoses, metabolic risk factors, regular somatic care, and routine check‐ups.

**Methods:**

This study used a 14‐item questionnaire to survey the somatic care situation of psychiatric university hospital patients. Main survey topics were psychiatric and somatic diagnoses, metabolic risk factors, regular somatic care, and routine check‐ups.

**Results:**

Four‐hundred and thirty‐five people with mental illness (48.3% male, mean age 45.4 years) were included. More than three quarters of the participating people with mental illness had access to a general practitioner. People with affective and anxiety disorders reported significantly more contact with medical specialists for somatic diseases, but schizophrenic patients did not receive enough care. Not all people with mental illness and on psychiatric medication received regular somatic care. Somatic diseases increased with number of diagnoses, and the duration of the psychiatric illness was positively correlated with treatment motivation.

**Conclusion:**

The observed unmet medical needs in this study might reflect the lack of treatment motivation in people with mental illness, but could also represent their obstacles to access care as well as a suboptimal communication between the treating psychiatrist and the referring general practitioner. Increasing awareness of somatic diseases in psychiatric patients and easier access to somatic care have to be implemented in psychiatric clinical routine. The risk of stigmatization in somatic institutions and the lack of self‐care management in people with mental illness are complicating factors.

## 1 INTRODUCTION

Mental illness is generally characterized by a combination of abnormal thoughts, perceptions, emotions, behavior, and relationships with others.^1^ Mental illness is an important public health problem, both in its own right and because it is associated with other chronic diseases and their resulting morbidity and mortality. According to the World Health Organization (WHO), mental illness accounts for more disability in developed countries than any other illness, including cancer and heart disease.^2^

Compared to the general population, individuals with mental illness such as schizophrenia, major depression, or bipolar disorder have worse physical health and reduced life expectancy.^3^, ^4^, ^5^ They die up to 20 years younger than people without mental illness^6^ and the mortality gap between people with mental illness and the general population seems to widen further over time.^7^, ^8^ This is not only true for people with so‐called severe mental illness but also for people with “common” mental disorders (such as substance use disorders, depression, and anxiety), since their risk for, for example, cardiometabolic diseases is elevated as well.^5^ According to Laursen et al, all findings point to four different possible reasons for the increase of premature deaths among people with mental illness: suboptimal lifestyles, adverse effects of antipsychotics, risk of suicide or accidents, and the late or insufficient treatment of somatic diseases.^9^ The proportion of psychiatric patients not receiving routine somatic monitoring to assess metabolic risk factors, even for those that are easy to apply (eg, obesity and blood pressure), is high.^10^, ^11^ Additionally, despite clear guidelines and high prevalence of undiagnosed diabetes mellitus, screening rates for metabolic abnormalities in psychiatric patients remain low, and up to 70% of people taking antipsychotics remain unscreened and untreated.^7^, ^12^ Although annual screenings for cardiovascular and metabolic disorders could be cost effective, people with mental illness are also less likely than people without mental illness to have a primary care record of cardiovascular conditions.^13^, ^14^

It seems that the somatic well‐being of people with mental illness has been neglected for decades^15^ and chronic somatic diseases seem to be under‐treated or under‐detected among this group.^9^ Some people with mental illness might also have poor knowledge about physical activity, dietary habits, and chronic physical problems^16^, ^17^ and might therefore prioritize their physical needs differently and exhibit different levels of motivation to change high‐risk behavior.^18^ Owing to their illness and stigmatization, people who suffer from mental diseases may have limited access to health services or experience poorer quality medical care, including promotion and prevention, screening, and treatment, than would be expected in the general population. Also, the lack of consensus about who should take responsibility for the general healthcare needs of people with mental illness may result in a continuing failure to provide appropriate services.^18^ Thus, the confluence of patient, provider, and systemic factors has created a situation in which access to and quality of health care is problematic for people with mental illness.^19^

The present study aimed to investigate the somatic status of this group with a focus on somatic diagnoses, metabolic risk factors, regular somatic care (by general practitioners and specialists), and routine check‐ups. An additional survey among a focus group used a more elaborate questionnaire for the detailed investigation of somatic health problems as well as the identification of perceived barriers in healthcare access.

## 2 MATERIAL AND METHODS

### 2.1 Procedure

This cross‐sectional observational study was performed on 435 in patients and outpatients of a psychiatric university hospital (mean age 45.4 years, 48.3% male). The study was approved by the Medical Ethics Committee of the Ruhr University Bochum (4392‐12), and all participants provided written informed consent. During the study period of 6 months, all patients who attended hospital treatment were asked about their current somatic care situation. Inpatients were contacted during the day on their respective ward, and outpatients were asked in the waiting room before seeing the therapist. Patients' psychiatric diagnoses were confirmed by the treating and trained psychiatrist. All patients were markedly ill with recurrent episodes of a chronic psychiatric disease. Subsequently, a focus group was recruited from the total sample, consisting of 89 patients who consented to be interviewed for further and detailed investigation (mean age 41.4 years, 44.7% male).

### 2.2 Measures

A paper‐based questionnaire comprising 14 items was prepared to assess personal information, smoking habits, height, weight, primary and background diagnoses, and current medication. Four major risk factors for cardiovascular diseases (arterial hypertension, diabetes mellitus, obesity, smoking) were assessed dichotomously and a corresponding risk score (0‐4) was calculated. Participants were asked whether they visited a general practitioner (GP) and informed them about their psychiatric condition. Specific items dealt with the regular use of the medical health system and routine check‐up procedures (blood test, electrocardiogram (ECG)). Moreover, the number of visits to the GP in the last year as well as date of last visit was inquired. The focus group completed 54 additional items covering specific aspects of their healthcare, further sociodemographic variables (migration background, marital status, occupational status, substance abuse), and a visual analog scale (VAS) for pain. Four additional dichotomous questions in the style of the Morisky Medication Adherence Scale^20^ were formulated to assess participants adherence to their prescribed medical regimen: “Vergessen Sie manchmal, Ihre Medikamente zu nehmen?“, „Sind Sie manchmal sorglos beim Einnehmen der Medikamente?,” “Wenn Sie sich besser fühlen, nehmen Sie dann manchmal keine Medikamente?,” “Wenn Sie sich manchmal nach der Einnahme der Medikamente schlechter fühlen, hören Sie dann auf diese einzunehmen?” “Do you forget sometimes to take medication?, Are you sometimes without sorrows in taking medication?, In case you feel better, are you then tending to take no medication?, In case you feel worse after medication, is it possible that you then stop to take them?.” Agreement with one of these statements scored one point. and a corresponding medication adherence score (0‐4) was calculated. A maximum score of four points would indicate the lowest adherence to prescribed medication. Furthermore, participants were asked for treatment adherence using a subsection consisting of 16 items rated on a five‐point Likert scale ranging from “always” to “never,” including questions about the participants' treatment motivation, the perceived information provided by their practitioners, and the degree to which they were able to cope with their disorder. A total score for the participants' overall treatment motivation was generated by summing up the 13 items depicting treatment motivation within the subsection (possible range = 0‐52), while the three remaining items (“Other people in the doctor's waiting room stare at me pejoratively.”; “I cannot afford to pay the quarterly fee for medical health care.” “I experience my commute to the doctor's office as burdensome for various reasons.”) were observed seperately.

### 2.3 Data analysis

Statistical analysis was performed with SPSS for Windows software (IBM). To analyze the study cohort, descriptive statistical methods were used. Quantitative data were presented as mean and standard deviation and categorical data with number of subjects and percentage. For comparison of categorical and continuous variables, *t*‐tests, Chi‐squaretests, and variance or linear regression analyses were used where appropriate. Correlational analyses using Pearson's *r* for continuous and Kendall's *τ* for categorical variables were performed to further investigate the relationship between psychiatric variables (number of psychiatric diagnoses) and the amount of healthcare (last contact with general practitioner, last blood sample taken, last ECG) provided to the participants as well as their relationship with other somatic variables (risk profile for cardiovascular diseases, number of somatic diagnoses) with age and gender as control variables. Analysis of variance (ANOVA) was used in order to identify possible group differences for gender, age, and psychiatric diagnoses on the number of risk factors, number of somatic diagnoses, number of psychiatric diagnoses, last contact, last blood sample and last ECG. A *P*‐value of less than .05 was interpreted as significant. We corrected for multiple testings when necessary by using Bonferroni‐corrected levels of significance.

## 3 RESULTS

### 3.1 Morbidity and risk factors

The most common (primary and secondary) psychiatric disorders in the sample according to the International Classification of Diseases (ICD‐10) are affective (54.9%), psychotic (25.1%), substance use (18.6%), and personality (13.56%) disorders. Nearly two‐thirds of the participants (65.1%) reported a somatic condition with 1.41 somatic diagnoses on average (SD = 1.44). Mean weight was 81.7 kg (SD 19.8) and mean height was 172.6 cm (SD 18.5); 53.1% of the participants smoked on a regular basis, about one third (29.4%) had a BMI of 30 or above, one fifth (22.1%) suffered from arterial hypertension, and 6.9% had diabetes mellitus. Complete data on their risk profile was available from 383 participants. Eighty‐four participants did not exhibit any of the above‐defined risk factors, whereas four participants presented all of them. The majority of participants (*n* = 196, 51.2%) presented smoking as the sole risk factor; 78.2% of the participants reported having a general practitioner, of which 81.6% knew about their psychiatric diagnosis. Participants with affective (*x* ^*2*^ *=* 7.15, *P* = .007) and anxiety (*x* ^*2*^ *=* 5.29, *P* = .002) disorders reported significantly more contact with medical specialists for somatic diseases, compared to other diagnostic subgroups. Participants with schizophrenic disorders had more difficulties to regularly visit their GP (*x* ^*2*^ *=* 4.66, *P* = .03).

### 3.2 Somatic and psychiatric interaction

**FIGURE 1:**
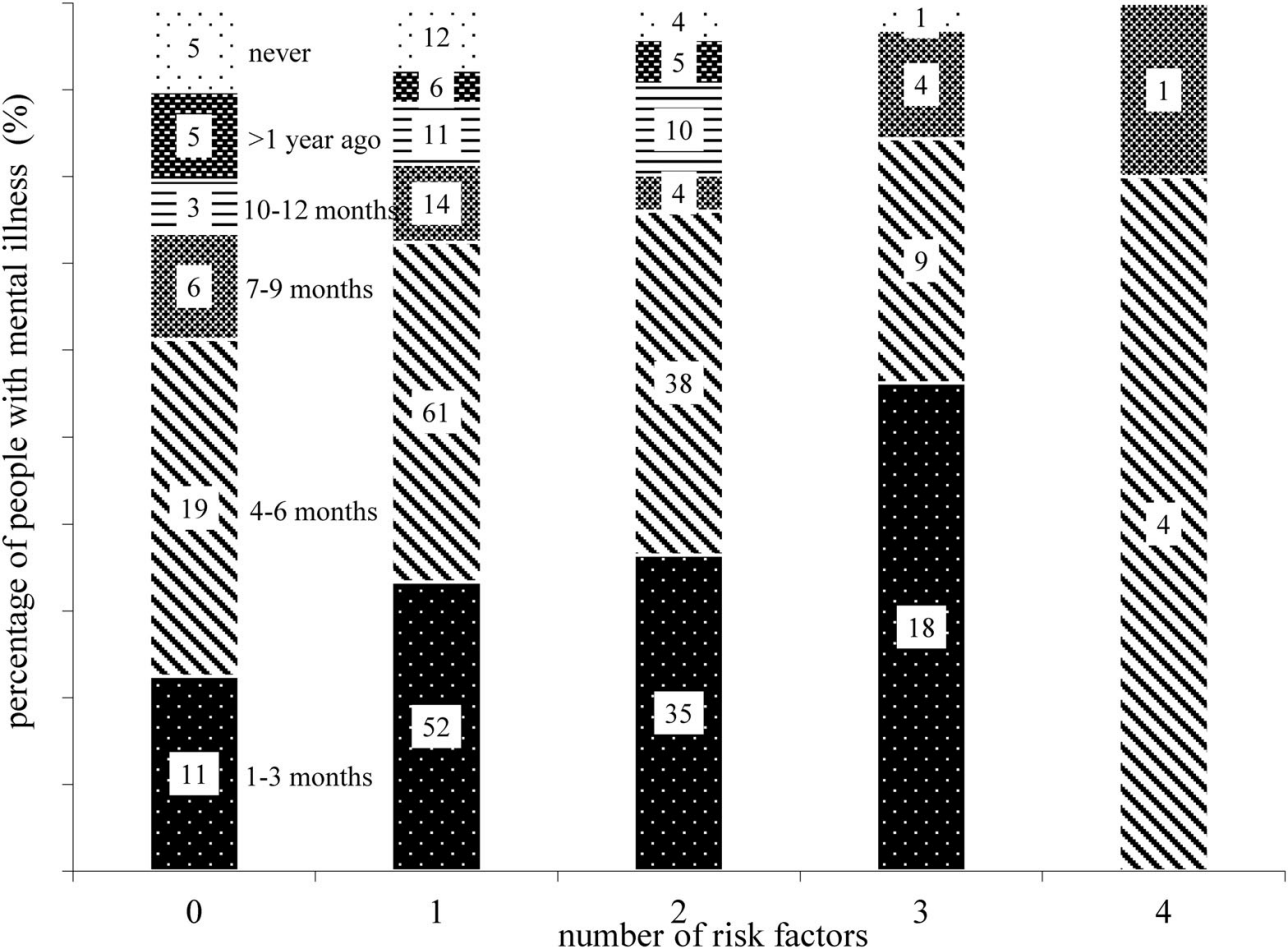
Risk factors and time interval since the last GP contact

The distribution of routine check‐up procedures as a function of the risk factors is depicted in Figure 1. About 10% of the participants with at least three risk factors reported that they never had a blood test and 20% had never had an ECG before. About 20% of the participants with four risk factors stated that their last blood test dated back more than a year. Table 1 shows the results of the correlational analysis. The number of somatic diagnoses was moderately positively correlated with age and the number of risk factors. Furthermore, we conducted analyses to identify possible relationships between ICD clusters and somatic variables. Psychotic participants seemed to be significantly under‐treated as measured by last blood sample (*τ* = 0.19, *P* = .000)and last ECG (*τ* = 0.28, *P* = .000). ANOVA yielded no significant results.

**TABLE 1:** Results of the correlational analysis

	Last GP contact	Last blood test	Last ECG	psychiatric diagnosis	somatic diagnoses	Risk factors	Age	Gender
Last GP contact	1.00	0.37**	0.25**	−0.08	−0.19**	−0.15**	−0.04	0.03
Last blood test		1.00	0.54**	−0.05	−0.21**	−0.09	−0.14	−0.05
Last ECG			1.00	0.01	−0.21**	−0.03	−0.20**	−0.05
Psychiatric diagnoses				1.00	0.07	−0.04	−0.17**	0.01
Somatic diagnoses					1.00	0.35**	0.34**	0.08
Risk factors						1.00	0.15**	0.00
Age							1.00	0.06
Gender								1.00

*Note*: Depicted are pearson's *r* for continuous and Kendall's *τ* for categorical variables.

^**^ Significant at *P* < .001.

### 3.3 Focus group

The majority of these participants (70.6%, *n* = 61) were living without a partner, and 14.1% (*n* = 12) of the them reported having no educational degree. Participants reported *M* = 2.51 (SD = 1.53) somatic and *M* = 1.66 (SD = 0.85) psychiatric diagnoses on average. The mean medication adherence score was *M* = 1.05 (SD = 1.24), indicating a medium level of adherence. Of the participants, 43.5% (*n* = 37) reported that they were suffering from acute pain with a mean VAS of *M* = 5.79 (SD = 2.65), indicating a medium level of pain. Treatment motivation observed was also medium, with *M* = 30.52 (SD = 6.80), but covered a large range (0‐52). Correlational analysis identified a positive correlation for age and number of risk factors (*r* = .16, *P* = .003), age and amount of somatic medication (*r* = .34, *P* = .003), as well as age and number of somatic diagnoses (*r* = .34, *P* = .000). There was also a positive correlation between Morisky score and the last blood test (*τ* = 0.32, *P* = .005) and a negative correlation between low medication adherence and participants' treatment motivation (*r* = −.27, *P* = .049). The duration of the psychiatric illness was positively correlated with treatment motivation (*r* = .30, *P* = .02) and last ECG taken (*r* = .32, *P* = .008). The amount of psychiatric medication was positively correlated with the number of risk factors (*r* = .42, *P* = .000) and the amount of somatic medication (*r* = .32, *P* = .004). The amount of somatic medication was also positively correlated with the number of risk factors (*r* = .45, *P* = .000). The perceived burden to reach the doctor's office was positively correlated with the duration of the mental illness (*r* = .30, *P* = .02) but there was no correlation with variables connected to somatic problems. There was no significant relationship between treatment motivation and number of risk factors, and the number of psychiatric diagnoses did not correlate with any other item. There was no correlation between the amount of psychiatric medication and the duration of the mental illness or the number of psychiatric diagnoses. Linear regression analysis with the number of risk factors as a predictor could not significantly predict treatment motivation or medication adherence. ANOVA did not yield any significant effect of the different levels of risk factors on treatment motivation, treatment adherence, and variables of medical care (last GP contact, last ECG, last blood sample).

## 4 DISCUSSION

In the present study, we investigated the somatic care received by a representative sample of people with mental illness in a German university hospital. Even though nearly all the participants were on psychiatric medication, some had never received a blood test or ECG by their own account. Participants especially seemed to feel discriminated against or stigmatized during their visits to their general practitioner. This is more alarming because psychopharmacological treatment often requires close medical examination. We observed significant negative correlations between the number of somatic diagnoses and the time spans since the last GP contact, the last blood sample, and the last ECG, indicating that severe somatic conditions led to proper somatic care depicted by shorter time intervals since the last medical procedures with increasing numbers of somatic diagnoses.

The lower the medication adherence, the longer the time span since the last diagnostic test. A low medication adherence also correlated negatively with the participants' treatment motivation, underlining the importance of both variables within the context of the treatment of mental disorders. Medication adherence is known to contribute to less functional outcomes considering the course of mental disorders such as frequent substance use or higher hospitalization rates.^21^ A better medication adherence could therefore be important to improve the somatic care situation of people with mental illness as well.

The observed unmet medical needs in this study might reflect the participants' lack of treatment motivation, but could also represent their obstacles to patient care access as well as a suboptimal communication between the treating psychiatrist and the referring GP. Whatever the reason, previous studies on mortality among people with mental illness have seen increased levels of mortality due to a lack of healthcare or its insufficiency.^9^ People with mental illness have been reported to suffer from the way other patients looked at them in their GP's practice. Stigmatization of mental disorders is known to prevent help‐seeking behavior among those in need.^22^ Psychiatrists and primary care physicians should play a more active role in ensuring that people with mental illness are not disadvantaged. There is a need to incorporate the screening, monitoring, and management of cardiovascular risk factors and diabetes in the psychiatric hospital routine to improve the overall health and well‐being of people with mental illness. As stated in the Lancet's blueprint for physical health care in people with mental illness, the development of integrated care models for efficient management of physical‐mental multimorbidity is crucial.^5^ It would be helpful to access the baseline risk for cardiovascular diseases at initial psychiatric presentation to monitor any subsequent change during treatment. The standard somatic system relies on a person's ability to initiate first contact with healthcare providers.^23^

In interpreting the results of this study, the following limitations should be considered Our analysis was based on self‐reported retrospective data, so the findings are subject to recall bias, and under‐ or over‐reporting of health care utilization might occur.^24^, ^25^ Information on the number of GP visits, medication, diagnoses, and treatment within the last 12 months was collected from patient questionnaires, which might have led to distorted estimation of the general medical care utilization. No further diagnostic procedures such as blood tests or ECG were performed to substantiate potential diseases or risk factors. Participants in the present study mainly suffered major depression. In comparison to people with other psychiatric disorders such as schizophrenia, bipolar disorder, or substance use disorder, they often receive less antipsychotic medication (as a major risk factor for metabolic and cardiovascular diseases) and are supposed to have a higher intrinsic level of using the healthcare system. Moreover, the specific German healthcare system differs from those in other countries, and results might therefore be not fully transferable.

Fortunately, one main finding from the present study is the improvement of care (at least in quantity) with increasing degrees of somatic illness, as indicated by the negative correlation between the number of somatic diagnoses and the time spans since the last blood sample, last ECG, and last GP contact. Still, the need for better diagnosis, quality treatment, and care strategies for somatic comorbidity in people with mental disorders is obvious. For further replication of our findings, it would be important to use prospective analysis using data from healthcare providers.

## CONFLICT OF INTEREST

The authors declare no conflict of interest.

## AUTHOR CONTRIBUTIONS

Conceptualization: Ida Sibylle Haussleiter, Georg Juckel

Formal Analysis: Barbara Emons, Knut Hoffmann

Resources: Ida Sibylle Haussleiter, Barbara Emons, Knut Hoffmann, Georg Juckel

Writing—Original Draft Preparation: Ida Sibylle Haussleiter

Writing—Review & Editing: Georg Juckel, Barbara Emons, Knut Hoffmann

 All authors have read and approved the final version of the manuscript.

 Georg Juckel had full access to all of the data in this study and takes complete responsibility for the integrity of the data and the accuracy of the data analysis.

## TRANSPARENCY STATEMENT

The corresponding author, Prof. Dr. Georg Juckel, confirms that this “manuscript is an honest, accurate, and transparent account of the study being reported; that no important aspects of the study have been omitted; and that any discrepancies from the study as planned (and, if relevant, registered) have been explained”.

## Data Availability

Data available on request from the authors.
